# Therapeutic Mechanisms of Berberine to Improve the Intestinal Barrier Function *via* Modulating Gut Microbiota, TLR4/NF-κ B/MTORC Pathway and Autophagy in Cats

**DOI:** 10.3389/fmicb.2022.961885

**Published:** 2022-07-22

**Authors:** JingWen Cao, MiaoYu Chen, Ran Xu, MengYao Guo

**Affiliations:** ^1^Department of Clinical Veterinary Medicine, College of Veterinary Medicine, Northeast Agricultural University, Harbin, China; ^2^Department of Clinical Veterinary Medicine, College of Veterinary Medicine, Huazhong Agricultural University, Wuhan, China

**Keywords:** inflammatory bowel disease, gut microbiota, intestinal barrier function, berberine, TLR4/NF-κB signaling pathway, autophagy, mTOR complex

## Abstract

**Background:**

Inflammatory bowel disease (IBD), a disease that seriously harms human and animal health, has attracted many researchers’ attention because of its complexity and difficulty in treatment. Most research has involved rats and dogs, and very little was cats. We should know that gut microbiota varies significantly from animal to animal. Traditional Chinese Medicine and its monomer component have many advantages compared with antibiotics used in pet clinics. Numerous studies have shown berberine (berberine hydrochloride) therapeutic value for IBD. However, the specific mechanism remains to consider.

**Results:**

We assessed gut pathology and analyzed fecal bacterial composition using Histological staining and 16S rRNA sequence. Dioctyl sodium sulfosuccinate (DSS) administration destroyed intestinal mucosal structure and changed the diversity of intestinal flora relative to control. RT-PCR and western blot confirmed specific molecular mechanisms that trigger acute inflammation and intestinal mucosal barrier function disruption after DSS treatment. And autophagy inhibition is typical pathogenesis of IBD. Interestingly, berberine ameliorates inflammation during the development of the intestinal by modulating the toll-like receptors 4 (TLR4)/nuclear factor kappa-light-chain-enhancer of activated B cells (NF-κB) signaling pathway and activating autophagy. Berberine significantly reduces tumor necrosis factor α (TNF-α), interleukin (IL)-6, and IL-1β expression in cats’ serum. Enhancing the antioxidant effect of IBD cats is one of the protective mechanisms of berberine. We demonstrated that berberine repairs intestinal barrier function by activating the mammalian target of rapamycin (mTOR) complex (MTORC), which inhibits autophagy.

**Conclusion:**

Berberine can restore intestinal microbiota homeostasis and regulate the TLR4/NF-κB pathway, thereby controlling inflammatory responses. We propose a novel mechanism of berberine therapy for IBD, namely, berberine therapy can simultaneously activate MTORC and autophagy to restore intestinal mucosal barrier function in cats, which should be further studied to shed light on berberine to IBD.

## Introduction

Cats are the most popular pets nowadays (Crowley et al., 2020). The change of living environment led to the change of diet from high protein to high carbohydrate (Grzeskowiak et al., 2015); it is one of the essential factors that predispose domestic cats to gastrointestinal diseases (Makielski et al., 2019). The fact that the gut is known as the “second brain” speaks volumes about the importance of a healthy gut. However, treating gastrointestinal diseases has always been a challenge for humans and pets.

Inflammatory bowel disease (IBD) is a common gastrointestinal disorder in cats and is a chronic immune-mediated disease affecting the gastrointestinal tract; so far, it is incurable (Marsilio et al., 2021). The number of IBD patients increases year by year due to wrong living habits (Habtemariam, 2016). Many studies have investigated the therapeutic agents of intestinal flora on IBD (de Mattos et al., 2015; Levine et al., 2018). Furthermore, intestinal barrier dysfunction was an essential mechanism of IBD, which has attracted more and more researchers’ attention (Glassner et al., 2020).

Antibiotics using in IBD, but whether they affect the body for better or worse remains highly controversial (Ianiro et al., 2016). To that end, researchers are turning to natural medicines that are safe and effective. Berberine hydrochloride (BBr) is the main ingredient in many commonly used Traditional Chinese Medicines (e.g., the genus Berberis). Studies have shown that BBr displayed numerous pharmacological activities, including anti-inflammatory as a mechanism of action for its therapeutic use in treating IBD (Chen et al., 2013, 2014; Habtemariam, 2016). However, the interplay between BBr and IBD is far more complex than it has to articulate.

We hypothesized that BBr would be safe and effective for IBD prevention. Here, we established IBD models in American shorthair treated with 5% dioctyl sodium sulfosuccinate (DSS) for 7 days to compare the clinical and histological changes with or without administration and further explore the therapeutic mechanism of BBr. In the following, we present data that demonstrates BBr restored intestinal barrier function, maintained intestinal microbiota homeostasis, regulated the expression of toll-like receptors 4 (TLR4)/nuclear factor kappa-light-chain-enhancer of activated B cells (NF-κB), and activated both the mammalian target of rapamycin (mTOR) complex (MTORC) and autophagy. Thus, it suggests that the therapeutic mechanism of BBr is remodeling intestinal flora, restoring intestinal barrier function, alleviating inflammation through modulation of the TLR4/NF-κB pathway, and preventing intestinal mucosal over-repair by activation of MTORC and autophagy.

## Materials and Methods

### Animal

This experiment conforms to the Animal Care and Use Committee of Northeast Agricultural University (SRM-11). The present study used twelve healthy adults American Shorthairs, including six women (all intact) and six men (all intact). They were vaccinated with the triple vaccine and were not in the vaccination period at the experiment. *In vitro* deworming was performed once a month, and *in vivo* deworming was performed every 3 months to eliminate the effects of viruses and parasites.

### Drug Administration

**FIGURE 1:**
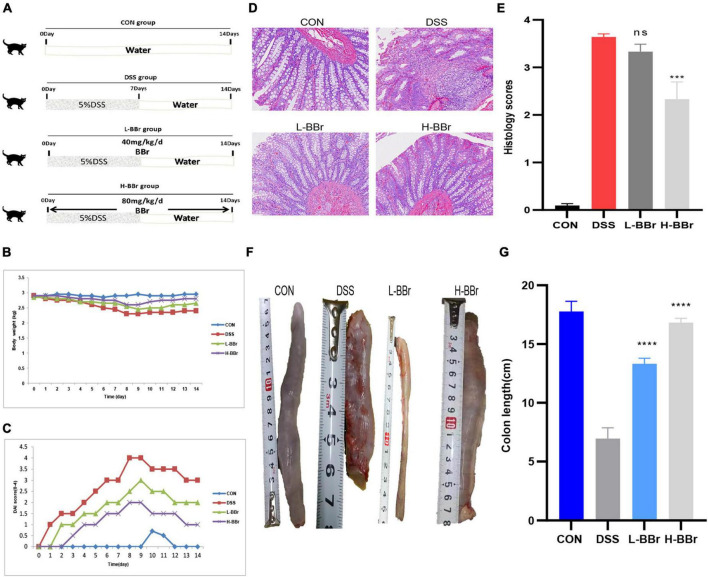
BBr exerted protective effects in DSS-induced IBD. **(A)** Graphical presentation of the study design. Intestinal inflammation is induced by administrating 5%DSS for 7 days and then replaced with pure water. Cats in the BBr group received BBr (40 and 80 mg/kg) orally for 14 days, and the vehicle group was left untreated. Bodyweight **(B)** and DAI score **(C)** monitor every day. **(D)** HE stained. **(E)** Colonic mucosal damage score. **(F)** Representative necropsy photographs of the colon. **(G)** Colon length. All data are present as mean ± *SD*. *P* < 0.05 was considered statistically significant, and ***P < 0.001, ****P < 0.0001 vs. the DSS group. CON, the control group, cats with no treatment; DSS, cats treated only with 5%DSS; L-BBr, cats treated with both 5%DSS and 40 mg/kg/day BBr; H-BBr, cats treated with both 5%DSS and 80 mg/kg/day BBr.

All cat adaptability raised a week with 12 h of light/12 h of the dark cycle, 25°C, and free access to food and water. Cats allocate to three groups: control (CON or A, *n* = 3), vehicle (DSS or B, *n* = 3), and BBr-treated vehicle group (*n* = 6; BBr, Figure 1A). To verify the effect of BBr dose on curative effect, six cats in the BBr group divide into two groups: the low-dose group (C or L-BBr, 40 mg/kg) and the high-dose group (D or H-BBr, 80 mg/kg). A total of 5% DSS (w/v, DSS, 36–50 kDa, MP Biomedical, Solon, OH, United States) was added to the drinking water over 7 days to induce experimental IBD and then replaced with normal drinking water for an additional 7 days (Figure 1A). BBr (Sigma-Aldrich, St. Louis, MO, United States) was administered orally at 40 and 80 mg/kg daily to the BBr group for 14 days (Figure 1A). Cats have euthanized on Day 14 for the following analysis.

### Fecal Collection

Feces have collected from each cat within 5 min of 2 ml glycerin injected into the rectum with a 2 mm diameter rectal administration tube after drug administration (day 14) and frozen at −80°C until further analysis.

### Clinical Monitoring and Determination of the Histological Score

During the process of IBD, experienced personnel determined the cat IBD activity index score (DAI) as previously reported (Jergens et al., 2010) to assess the severity of inflammation in colitis, including bodyweight loss, stool consistency, and fecal blood (Supplementary Table 1).

On Day 14, Cats were euthanized and measured the length of the colon; about 1 cm of colon tissue was removed and fixed in 10% formalin to stain with H&E. The extent of tissue damage was observed under Leica laser microdissection systems (DM6B, Heidelberg, Germany) and assessed in Supplementary Figure 1 (Oktar et al., 2002). Performed all injury assessments by observers unaware of the treatment and expressed the final score as an average.

### Oxidative Stress Measurement

The experiment examined only three groups with significantly different antioxidant levels: the CON, DSS, and H-BBr groups. Normal saline was mixed in 1 *g* of colon tissue from each group, ground on ice to homogenate, and taken the supernatant after centrifugation, determined the protein concentration with a BCA protein determination kit (Mei5 Biotechnology Co., Ltd, Beijing, China). The contents of CAT, T-AOC, GSH, MDA, and SOD in colon homogenate were detected using a CAT, T-AOC, GSH, SOD, and MDA assay kit (Njjcbio, Nanjing, China) following the manufacturer’s instructions.

### 16S rRNA Sequence Analysis

We used a 16S rRNA sequence to analyze the effects of BBr on the communities of intestinal flora and extracted total DNA from 2 *g* of feces in each group. SEQHEALTH (Wuhan, China) conducted the sequencing work. Nanodrop was used to quantify DNA and detected the extracted DNA quality by 1.2% agarose gel electrophoresis. We used the V3 + V4 region of the 16S rRNA gene, the target for PCR amplification. The amplified products were recovered and quantified by fluorescence. The sequencing library should be prepared using the TruSeq Nano DNA LT Library Prep Kit (Illumina, San Diego, CA, United States) and performing high-quality sequencing. For this experiment’s amplicon sequencing bioinformatics workflow, as follows:

The raw sequence is divided into library and sample according to index and Barcode information and then removed the Barcode sequence. Sequence denoising or OTU clustering was performed according to the QIIME2 DADA2 analysis or Searched software analysis process. Presented the specific composition of each sample (group) at the taxonomic level of different species to understand the overall situation. According to the distribution of ASV/OTU in different samples, each sample evaluated the Alpha diversity level and reflected the appropriate sequencing depth through the light curve. We calculated each sample’s distance matrix at the ASV/OTU level. At the level of taxonomic composition, we further measured the diversity of species abundance composition among different samples (groups) through various unsupervised sorting, clustering, and modeling methods, combined with corresponding statistical test methods, and tried to find marker species.

### Enzyme-Linked Immunosorbent Assay

The forelimb saphenous vein blood was collected on the 14th day of treatment and centrifuged at 15,000 RPM/min for 15 min to separate the serum. The serum freezing at −80°C for later use. According to the manufacturer’s instructions, the concentrations of interleukin (IL)-6, IL-1ß, and tumor necrosis factor α (TNF-α) in the serum sample detecting by the ELISA kit (Cell Signaling Technology, Boston, United States). We draw a standard curve to calculate the content of cytokines in the sample.

### RNA Extraction and Quantitative PCR

We conducted RT-PCR to verify gene-level changes in the CON, DSS, and H-BBr groups. Extracted total RNA from colon tissues using RNeasy Plus Kit (Invitrogen, New York, United States). Following the manufacturer’s instructions, reverse transcription performing by using the BioRT Master HisSensi cDNA First-Strand Synthesis kit (Shanghai Roche, China). PCR2720 thermal cycler (Applied Biosystems, United States) and ABI7500 PCR system perform RT-qPCR. The fluorescence reagent used in this experiment was SYBR Green I, and the cycling conditions refer to Supplementary Figure 2. The primers used for PCR amplification which listed in Supplementary Table 2.

### Western Blot

We detected the protein levels of the CON group, DSS group, and H-BBr group by using Western blot. Extracted tissue protein on ice, and the protein concentration was determined with a BCA protein determination kit and diluted to an equal concentration (2–4 μg). Protein was separated by 6, 8, 10, and 12% SDS-polyacrylamide gel electrophoresis. The membrane transfer time is proportional to the protein size. After sealing with 5% non-fat milk for 2 h at room temperature, the membrane was incubated overnight with cat primary antibody (Supplementary Table 3) at 4°C and acquired with peroxidase-conjugated secondary antibody goat anti-rabbit IgG (1:2,000, Cell Signaling Technology) for 2 h at room temperature. Finally, it detected the signal with X-ray films (TransGen Biotech Co., Beijing, China).

### Statistical

Using GraphPad Prism 7 software for experimental data, Student’s *T*-tests and one-way ANOVA analyze differences between groups. We processed the images by using Adobe Photoshop 2020 and Imagej_V1.8. SPSS 13 and statistics were analyzed by means ± standard deviation ($\bar{\text{x}}$ ± s). *P* < 0.05 was considered statistically significant.

## Results

### Berberine Hydrochloride Alters the Experimental Features in Dioctyl Sodium Sulfosuccinate-Induced Inflammatory Bowel Disease

All cats developed significant IBD symptoms following DSS uptake, as evidenced by a severe DAI score (Figures 1B,C) and colon shortening (Figures 1F,G). However, BBr reverses these clinical characteristics (Figures 1A–G). The histological examination demonstrated that BBr showed a significant protective effect on colon damage and inflammation, with complete morphology of epithelial cells and abundant goblet cells, complete repair of the glandular structure, but still a small amount of inflammatory cell infiltration (Figures 1D,E). Consistently, the expression of serum inflammatory factors was upregulated in DSS-treated, while IL-6, IL-1β, and TNF-α were down-regulated after BBr treatment (Supplementary Figure 3). Moreover, DSS exposure disrupts the antioxidant system in the gut (Li H. et al., 2020). Compared with the CON group, the content of MDA increased in the DSS group while it decreased in the H-BBr group (Supplementary Figure 4). Meanwhile, the H-BBr group had an increased antioxidant effect, as evidenced by increased T-AOC, GSH, CAT, and SOD values (Supplementary Figure 4). Notably, the greater the dose of BBr, the less pronounced the DSS-induced IBD features.

### Berberine Hydrochloride Rebuilt the Intestinal Tight Junction Barrier

**FIGURE 2:**
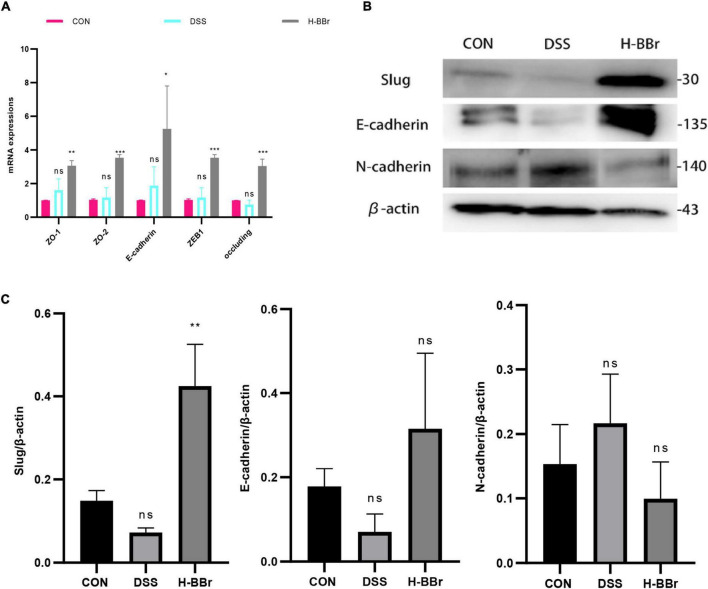
BBr repairs DSS – induced intestinal mucosal barrier injury. **(A)** Histogram of the mRNA relative expression in colonic mucosa. **(B)** Western blot assay of E-cadherin, N-cadherin, and slug. **(C)** One-way ANOVA and Tukey’s post-test analyze the protein gray value of E-cadherin, N-cadherin, and slug. Data represent the mean ± *SD*. *P* < 0.05 was considered statistically significant. *P < 0.05, **P < 0.01, ***P < 0.001 vs CON group.

The intestinal epithelium consists of intestinal epithelial cells and tight junction proteins, maintaining mucosal homeostasis (Parikh et al., 2019). We investigated the therapeutic effect of BBr on DSS-induced intestinal barrier dysfunction. We assessed the mRNA expression level of colonic tight junction proteins (ZO-1, ZO-2, E-cadherin, ZEB1, and occluding) by RT-PCR. As shown in Figure 2A, in contrast to the CON group, BBr significantly upregulated the mRNA expression level of tight junction proteins. Meanwhile, Western blot results demonstrated that BBr could repair the intestinal mucosal barrier. As illustrated in Figures 2B,C, increased E-cadherin and slug expression in H-BBr-treated cats and down-regulated the expression level of N-cadherin.

### Berberine Hydrochloride Modulates Dioctyl Sodium Sulfosuccinate-Induced Gut Microbiota Dysbiosis

**FIGURE 3:**
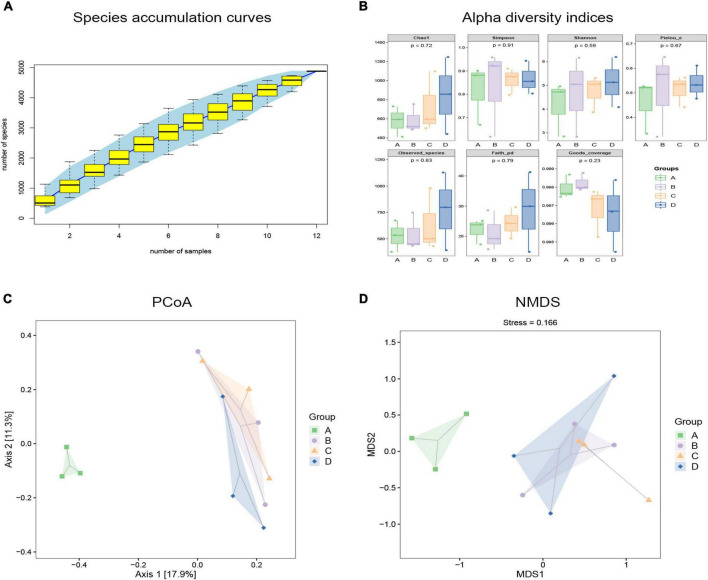
Analyze each group’s gut microbiota richness and diversity. **(A)** Specaccum species accumulation curve. The *X*-axis represents sample size, and *Y*-axis stands for the number of observed species (ASV/OTU). The blue part is the confidence interval of the curve (*n* = 3). **(B)** Alpha diversity indices of fecal samples in each group (*n* = 3). Principal coordinate analysis (PCoA; **C)** and non-metric multidimensional scaling (NMDS; **D)** based on OTU abundance (*n* = 3). The oval dotted circle represents the 95% confidence region. A: CON group; B: DSS group; C: L-BBr group; and D: H-BBr group.

To observe the effects of BBr on the gut microbiota, we evaluated the composition and diversity of intestinal microbiota using 16S rRNA sequence analysis. It can be seen in Figure 3A that the species accumulation curve of cat feces collected in this experiment tends to be smooth, which proves that the sequencing depth is adequate. To comprehensively assess the alpha diversity of the microbial community, the Chao1 and Observed Species index is used in this process to characterize the richness. The Shannon and Simpson index characterized diversity, and Faith’s PD index characterized evolution-based diversity. Pielou’s Evenness index characterized evenness, and Good’s Coverage index characterized coverage. Compared with the CON group, the diversity of the DSS group increased, but its richness and evolutionary diversity decreased (Figure 3B). As a carnivore, the diversity of intestinal flora of cats is much smaller than that of omnivores. The smaller the diversity of intestinal flora, the worse its stability. After DSS application, many harmful bacteria and opportunistic bacteria colonized, resulting in increased species diversity of the DSS group, but after BBr treatment, species diversity decreased (Figure 3B). Estimated differences in gut microbiota composition between groups by employing the principal coordinates analysis and non-metric multidimensional scaling. In the coordinate graph, species composition and structure differences are proportional to the distance between the two samples. The results showed little difference between the CON and H-BBr groups, while the composition and structure of intestinal flora in the DSS group were different from the other groups (Figures 3C,D). Taken together, BBr can partially alleviate the intestinal microflora disorder of IBD, and the effect is proportional to the dose.

**FIGURE 4:**
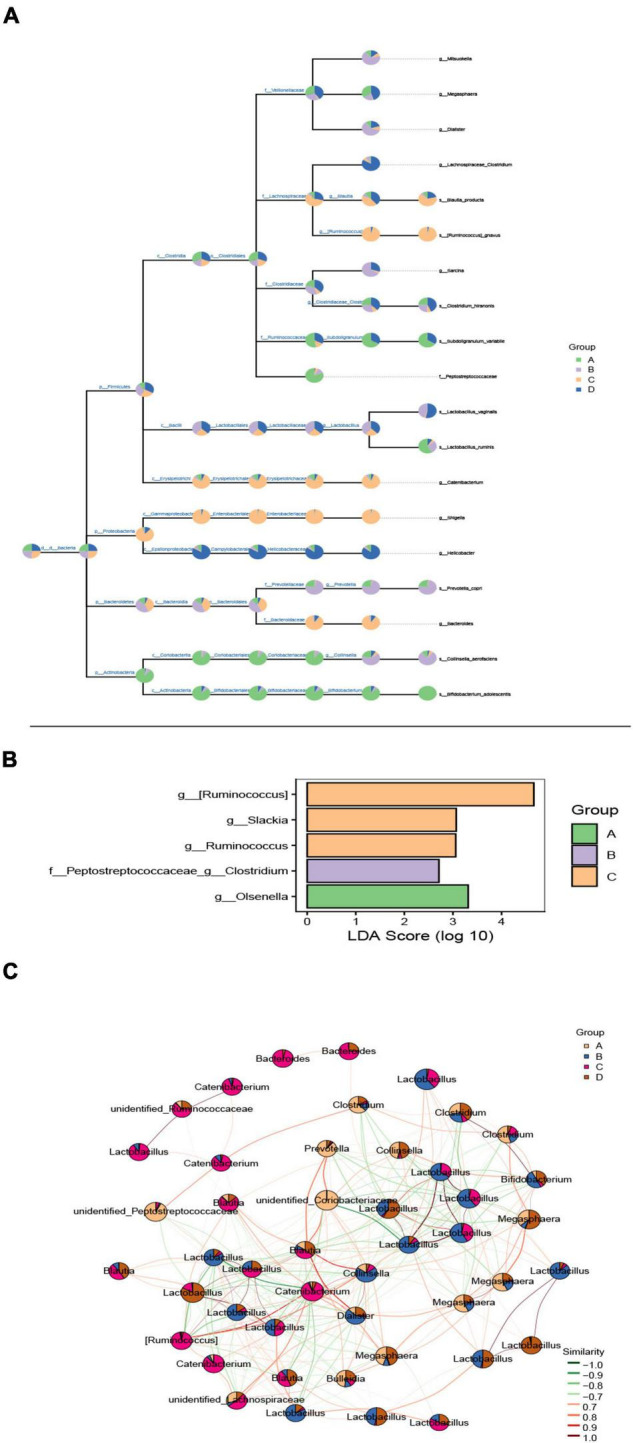
The effect of BBr on gut microbiota composition. **(A)** Classification hierarchy tree diagram. The pie chart (threshold 0.5%) of each branch node in the classification hierarchy tree shows each group’s composition proportion of the taxon. The fan-shaped area represents the abundance of bacteria, and there is a positive relationship between them. **(B)** Histogram of marker species’ LDA affects value. The *X*-axis represents the logarithmic score value of LDA analysis, which is higher between groups and is more different. *Y*-axis is the marker species. **(C)** Dominant seed network diagram. The node size is proportional to its abundance [log2 (CPM/N) as a unit]. The pie chart shows the relative abundance ratio in different groups. The edge line indicates a correlation between the two nodes connected. The red line indicates a positive correlation, and the green line indicates a negative correlation. A: CON group; B: DSS group; C: L-BBr group; and D: H-BBr group.

Based on the above results, we expected to determine the intestinal microflora taxa and their relative abundance to determine the exact species with significant differences between the two groups. Here, draw a microbial classification hierarchy tree and show the composition of all taxa. As shown in Figure 4A, Firmicutes, Bacteroidetes, Proteobacteria, and Actinobacteria were the phyla with a significant difference. Prevotella was the noticeably varied bacteria at the genus level in the DSS group, and the relative abundance of bacteria increased in the DSS compared to the CON group. Next, a heatmap figured out the exact species with a significant difference in the two groups. There were 50 species with significant variations and was a significant difference between DSS and CON groups (Supplementary Figure 5). LDA Effect Size analysis finds the marker species significantly different from each group. As illustrated in Figure 4B, Olsenella is the landmark species of the CON group, which belongs to a group of bacteria under the subclass Red Hemiptera of actinomycetes; the iconic species in the DSS group: Peptostreptococcaceae Clostridium is a bacterium belonging to the genus Clostridium of Digestive Streptococcaceae; the signature species of BBr group: Ruminococcus and Shrektonia.

Last but not the least, based on the network analysis of the relationships among microbial members, we try to explore the specific group of microorganisms to perform specific ecological functions and the keystone change is enough to move the composition of the entire community. As shown in Figure 4C, Lactobacillus was the most dominant species in the experimental group, and there was a negative correlation between BBr and DSS group.

### Functional Prediction of Intestinal Microbiota Community After Berberine Hydrochloride Treatment

**FIGURE 5:**
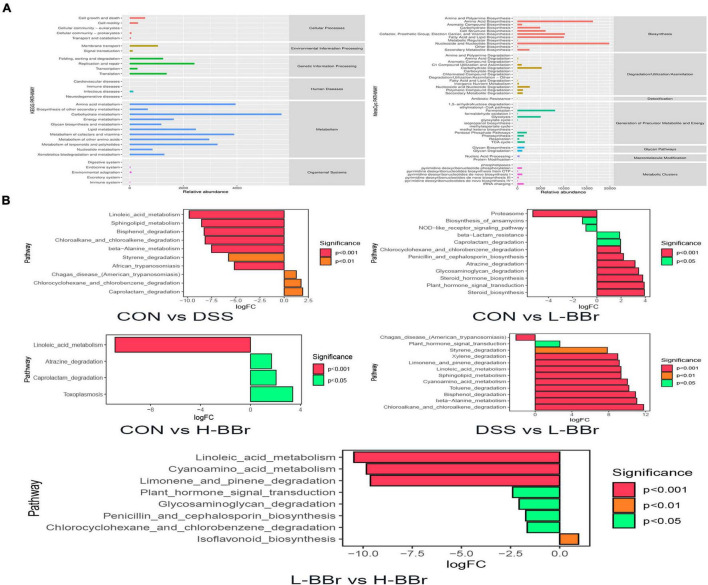
Potential biomarkers and correlation analysis. **(A)** The KEGG (left) and MetaCyc (right) functional pathway abundance map. The *X*-axis stands for relative abundance, and *Y*-axis represents function class. **(B)** The *X*-axis represents logFC[log2(fold change)], the positive value represents up-regulation of group **(B)** relative to group **(A)** the negative value is down-regulation, *Y*-axis stands for function class.

We employed KEGG and MetaCyc to analyze the metabolic and biosynthesis pathway and figure out the possible functions of intestinal microbiota after BBr treatment. As shown in Figure 5A, the metabolism and biosynthesis of amino acids have the highest abundance, followed by carbohydrate metabolism and nucleotide biosynthesis. Meanwhile, the abundance of species associated with fatty acid and lipid biosynthesis increased remarkably. Furthermore, we attempted to identify metabolic pathways with significant differences between groups. As illustrated in Figure 5B, after DSS treatment, the relative abundance of species whose functions are associated with the metabolism of linoleic acid and sphingolipid were significantly downregulated, whereas BBr treatment was upregulating. It indicates that BBr can control metabolic disorders.

### Berberine Hydrochloride Reduces Inflammation by Regulating the TLR4/NF-KB Signaling Pathway in the Colon

**FIGURE 6:**
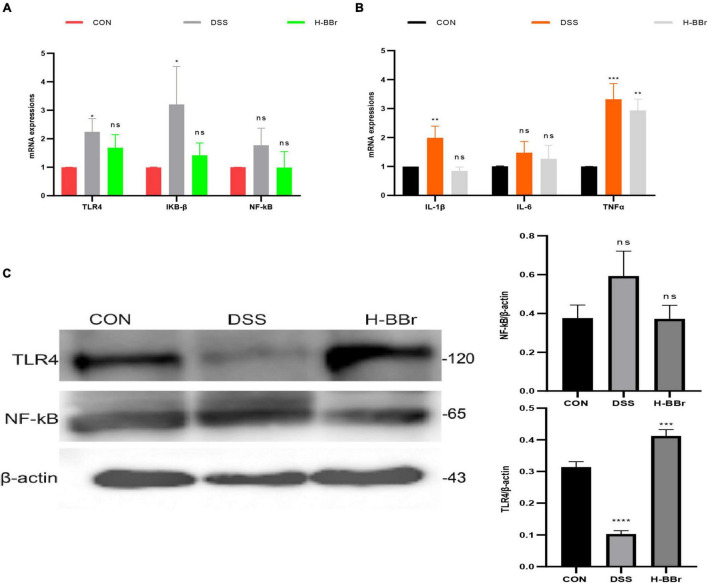
BBr reduces the expression of inflammatory signaling. mRNA expressions of TLR4/NF-kB signaling **(A)** and inflammatory factor (IL-1β, TNFα, and IL-6; **B)**. **(C)** Protein expressions of TLR4/NF-kB signaling pathway. Data represent the mean ± *SD*. *P* < 0.05 was considered statistically significant. *P < 0.05, **P < 0.01, ***P < 0.001, and ****P < 0.0001 vs. CON group.

Studies have demonstrated that TLR4/NF-KB signaling pathway is the key mechanism leading to persistent inflammation (Rathinam and Chan, 2018; Tang et al., 2021). TLR4/NF-KB signaling pathway was assayed in our research to understand the anti-inflammatory mechanism of BBr deeply. Compared with the CON group, the mRNA expressions of TLR4 and IKB-ß significantly increased in the DSS group (Figure 6A). More interestingly, protein levels of TLR4 were lower in the DSS group than in the other groups (Figure 6C). It should note that TLRs have both protective and harmful effects on inflammation (Rakoff-Nahoum et al., 2004). The host activates the defense system and controls TLR4 protein expression to avoid excessive uncontrolled inflammation. Meanwhile, BBr can inhibit the mRNA expression of IL-1β, TNFα, and IL-6 (Figure 6B).

### Berberine Hydrochloride Activates Both Mammalian Target of Rapamycin Complex and Autophagy

**FIGURE 7:**
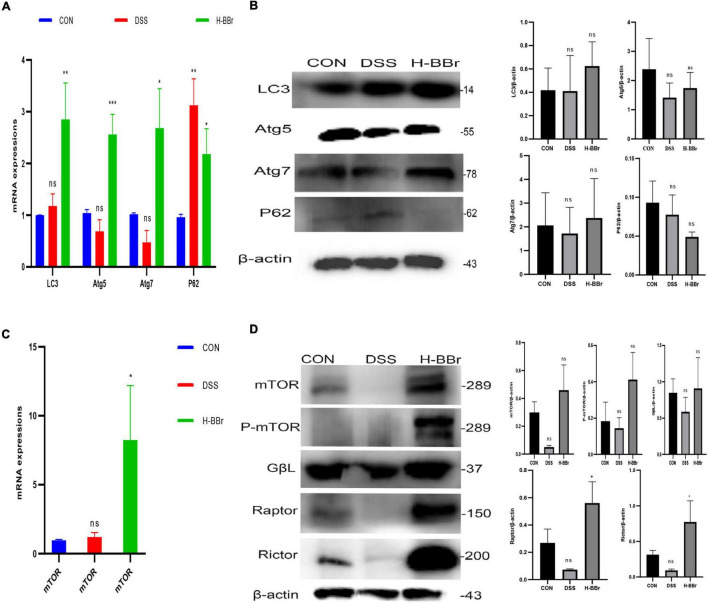
BBr-activated autophagy and MTORC are essential to cure colitis. mRNA **(A)** and Proteins **(B)** expressions of LC3, Atg5, Atg7, and P62. mRNA **(C)** and Proteins **(D)** expressions of MTORC (mTOR, Rictor, Raptor, and GβL) and P-mTOR. Data represent the mean ± *SD*. *P* < 0.05 was considered statistically significant. *P < 0.05, **P < 0.01, and ***P < 0.001 vs CON group.

The cellular process of autophagy is required to maintain intestinal homeostasis (Elshaer and Begun, 2017). BBr activated autophagy, as evidenced by the expression of autophagy indexes, such as LC3, Atg5, Atg7, and P62. As shown in Figures 7A,B, after BBr treatment, the expression of Atg5, Atg7, and LC3 proteins increased and downregulated the expression of P62. MTORC is a negative regulator of autophagy in IBD, composed of mTOR and several adaptors, including Raptor, Rictor, Raptor, and GβL (Kim and Guan, 2015; Cosin-Roger et al., 2017). In contrast, our study found that MTORC and P-mTOR activated in the BBr group (Figures 7C,D). Hence, the relationship between MTORC and autophagy is elusive and requires further study. To sum up, MTORC and autophagy are essential mechanisms of BBr in the treatment of IBD.

## Discussion

As the status of cats increased in the family, so did their health and welfare. Changes in the environment increase the risk of IBD in cats. Similarly, the number of people with IBD has increased over the years due to deviant lifestyles. Human IBD consists of 2 subtypes: Crohn’s disease (CD) and ulcerative colitis (UC) (Habtemariam, 2016). Despite being recognized for decades, IBD remains a clinical challenge today. Currently, IBD treating with antibiotics or immunosuppressant drugs. However, unwanted side effects and poor efficacy are inherent problems associated with these drugs (Ianiro et al., 2016). As a result, many traditional plant-based therapies have to explore as alternatives Moreover, the mechanism of Traditional Chinese Medicine on IBD has increasingly raised interest in recent years.

As one of the most representative and most studied natural alkaloids, BBr has been shown to display numerous pharmacological activities (Habtemariam, 2016). There has been a significant and increasing interest in exploring the IBD-preventive effects BBr during the last decades. Our results show that BBr reduces the expression of IL-1β, TNF-α, and IL-6 in serum, suggesting a reduced inflammatory response (Cosin-Roger et al., 2017). What is more, we found that BBr maintains the homeostasis of the antioxidant system in the gut (Parikh et al., 2019). Studies have shown that BBr can produce oxidized berberine (OBB) under the action of intestinal flora (Zhang et al., 2021), and OBB can significantly reduce colon shortening and histological damage in IBD patients (Li C. et al., 2020). Therefore, BBr may also interact with the intestinal flora of cats to generate OBB, which is anti-inflammatory, alleviates diarrhea symptoms of cats with colitis, and makes the intestinal microenvironment conducive to the colonization of good bacteria.

Abnormal homeostasis of intestinal flora is one necessary pathologic mechanism for IBD (Glassner et al., 2020). Our study based on 16S rRNA sequence analysis verified that the diversity and composition of intestinal flora in cats with IBD altered. In contrast to previous studies, the diversity of bacteria in this study increased after DSS treatment. The reason may be that the diversity of cats is lower than that of mice and dogs and disrupts the homeostasis more quickly. It should note that the DSS group had a single and small amount of bacteria. It further explains that DSS treatment can promote the propagation of harmful bacteria and temporarily cause an increase in bacterial diversity. We treated IBD cats with BBr and found that their gut microbiota was roughly the same as healthy cats. Firmicutes and Bacteroidetes are the major bacterial phyla in the gastrointestinal tract, and the Firmicutes/Bacteroidetes (F/B) ratio associating with maintaining homeostasis (Deng et al., 2022). Our results are consistent with previous studies showing a reduced F/B ratio in IBD cats compared to the CON group. However, Firmicutes and Bacteroidetes were more abundant than in the CON group. The increase of other phyla may influence the F/B ratio.

Significant differences in microbiota composition exist among species (Suchodolski, 2016; Stojanov et al., 2020). One should remember that changes in this ratio can cause ecological disorders, thereby leading to various pathologies. Here, we found that the F/B ratio increased after BBr treatment to that of healthy cats. Specific probiotics can restore the gut microbial balance by influencing the F/B ratio, as the genus Lactobacillus (Stojanov et al., 2020). Our results imply that BBr treatment dramatically increased the abundance of Lactobacillus, suggesting that certain probiotic strains can manage IBD. Nevertheless, the therapeutic effect of Lactobacillus is limited, and their proliferation can cause pathological damage (D’Alessandro et al., 2021; Liu et al., 2022).

A dysbiotic microbiome can affect the host not only directly but also indirectly by altering metabolic processes. Linoleic acid, a component of many cat diets, is degraded less in IBD cats, suggesting an abnormal digestive process that leads to weight loss in IBD cats. Notably, abnormal linoleic acid metabolism increases toxoplasma susceptibility (D’Alessandro et al., 2021). The abundance of Clostridium spp. is significantly reduced in IBD cats, whose primary function is to deconjugate bile acids and promote fat absorption in the small intestine (Suchodolski, 2016). However, certain beneficial bacteria (e.g., *Blautia* spp., *Faecalibacterium* spp.) that produce immune metabolites, such as SCFA increased in IBD cats, caused by the body’s defense mechanism (Suchodolski, 2016). It also illustrates that the intestinal tracts of cats harbor a highly complex microbiota.

Enterotoxins produced by pathogenic bacteria can destroy villous effacement and dysfunction the mucosal barrier (Suchodolski, 2016; Deng et al., 2022). The physical barrier of intestinal epithelial cells and tight junction proteins is part of the intestinal barrier (Stojanov et al., 2020). This study demonstrated that DSS increases intestinal paracellular permeability, alters tight junction proteins’ expressions, and destroys colon tissue morphology, suggesting that DSS induces intestinal tight junction barrier dysfunction *in vivo*. In line with the results obtained in patients with IBD, disrupted the intestinal barrier in cats with 5%DSS. Here, BBr treatment could improve the harsh pathological environments.

Intestinal microflora dysbiosis induces inflammatory reactions through TLRs (Suchodolski, 2016). When properly activated, Toll-like receptors protect the gut, but when overactivated, they can cause persistent inflammation that can lead to severe disease (Rakoff-Nahoum et al., 2004). TLR4, the best-characterized member of the toll-like receptors, is known to activate adaptive immune responses and upregulate the expression of inflammatory genes by activating the NF-kB pathway (Ciesielska et al., 2021). Notably, more expression of TLR4 in the colon in healthy cats (Asahina et al., 2003). It may explain why the protein level of TLR4 in the DSS group was lower than that in the other two groups. Hence, BBr can maintain intestinal function and repair the intestinal barrier by controlling TLR4/NF-kB pathway.

Dysfunction of autophagy is associated with the pathogenesis of CD (Larabi et al., 2020). Autophagy is an intracellular degradation pathway and an essential cell survival mechanism. The index of autophagy induction mainly includes microtubule-associated protein one light chain 3 (LC3), Atg7, P62, and Atg5 (Klionsky et al., 2021). We found that BBr treatment activated autophagy in IBD cats, as evidenced by increased LC3, Atg5, and Atg7 expression and inhibited P62 expression. Studies have suggested a tight, inverse coupling of autophagy induction and MTORC activation (Kim and Guan, 2015). The components of MTORC include Rictor, Raptor, and GβL (Szwed et al., 2021). We found that both autophagy and MTORC were activated in IBD cats with BBr treatment, suggesting that the cross-talk between autophagy and MTORC has contradictory results. A recent study has revealed autophagy-induced MTORC activation to avoid excessive myofibroblast accumulation (Bernard et al., 2020). Long-term stimulation of intestinal mucosa with DSS can induce chronic inflammation, typically characterized by excessive myofibroblasts accumulation. They were reversing this direct interaction under BBr treatment further. Taken together, BBr activates autophagy and MTORC to work together to repair the intestinal barrier.

In conclusion, the principal finding of this study is that BBr could severely recover intestinal microbiome homeostasis, including composition and function. More importantly, BBr reduces inflammation by inhibiting the colon’s TLR4/NF-KB signaling pathway. Furthermore, BBr activates MTORC and autophagy in the intestine to promote intestinal epithelial cell proliferation and maintain cell metabolism, restoring intestinal barrier function. These findings provide new insights into the mechanisms of BBr therapy for IBD, in which activated MTORC and autophagy may be involved. Indeed, understanding the composition of intestinal flora in cats is beneficial to finding targeted drugs to treat intestinal diseases in cats, which is of great significance for treating pet gastroenteropathy.

## Data Availability Statement

The raw data supporting the conclusions of this article will be made available by the authors, without undue reservation. The 16S rRNA sequencing data presented in the study are deposited in the NCBI repository, accession number PRJNA848954.

## Ethics Statement

The animal study was reviewed and approved by Animal Care and Use Committee of Northeast Agricultural University (SRM-11). Written informed consent was obtained from the owners for the participation of their animals in this study.

## Author Contributions

JC and MC: conceptualization and methodology. JC: writing—original draft and review and editing. JC, MC, and RX: data curation. All authors read and approved the final manuscript.

## Conflict of Interest

The authors declare that the research was conducted in the absence of any commercial or financial relationships that could be construed as a potential conflict of interest.

## Publisher’s Note

All claims expressed in this article are solely those of the authors and do not necessarily represent those of their affiliated organizations, or those of the publisher, the editors and the reviewers. Any product that may be evaluated in this article, or claim that may be made by its manufacturer, is not guaranteed or endorsed by the publisher.
