# Supplementary material for: Therapeutic Mechanisms of Berberine to Improve the Intestinal Barrier Function via Modulating Gut Microbiota, TLR4/NF-κ B/MTORC Pathway and Autophagy in Cats
Source: Front Microbiol. 2022 Jul 22;13:961885. doi: 10.3389/fmicb.2022.961885 (PMC9354406; doi:10.3389/fmicb.2022.961885)
Supplement: Supplementary file 1 [file Data_Sheet_1.docx]

**Table S1. Using independent variables is the developing.**

| **Score** | **Weight loss（%）** | **Stool consistency** | **Occult blood test** |
| --- | --- | --- | --- |
| 0 | None | Normal | Negative |
| 1 | 1-5 | Soft but still formed | Negative |
| 2 | 6-10 | Soft | Positive |
| 3 | 11-15 | Very soft; wet | Positive |
| 4 | ＞15 | Watery diarrhea | Fecal blood |
