# Supplementary material for: Therapeutic Mechanisms of Berberine to Improve the Intestinal Barrier Function via Modulating Gut Microbiota, TLR4/NF-κ B/MTORC Pathway and Autophagy in Cats
Source: Front Microbiol. 2022 Jul 22;13:961885. doi: 10.3389/fmicb.2022.961885 (PMC9354406; doi:10.3389/fmicb.2022.961885)
Supplement: Supplementary file 2 [file Data_Sheet_2.docx]

**Table S2. Sequences of primers for quantitative real-time PCR.**

| **Gene** | **Sequence 5′–3′** |  |
| --- | --- | --- |
| **Feline** |  |  |
| β-actin | F: CCTGGCACCTAGCACAATGA | R: CCTGCTTGCTGATCCACATC |
| TLR4 | F: GGCTGTGGAGACAAACAT | R: GTAAACCTGCCAGACCTT |
| NF-κB | F: GTTCGCATCTCATTGGTCAC | R: CGCATTCAGGTCGTAGTCCC |
| IKB-β  IL-1β  TNF-α  IL-6  mTOR  LC3  Atg5  Atg7  P62  ZO-1  ZO-2  E-cadherin  ZEB1  occluding | F: TGGCTCATCGTAGGGAGTTT  F: GTGGCTGTGGAGAAGCTGTG  F: TGTTGCTCCTTTCACTTCCAT  F: CCAGAAACCGCTATGAAGTTCC  F: TTGCTGTTGCCTCCTATCGTGAAG  F: CGTTGACATCCGTAAAGACCTCTA  F: AGTCAAGTGATCAACGAAATGC  F: GTGTACGATCCCTGTAACCTAG  F: GAACACAGCAAGCTCATCTTTC  F: AACCCGAAACTGATGCTGTGGATAG  F: CATGTCTCTAACGGATGCTCGGAAG  F: ACCAGCAGTTCGTTGTCGTCAC  F: AAGCCATACGAATGCCCGAACTG  F: TGGAGGCTATGGCTATGGCTATGG | R: CTCGTCCTCGACTGAGAAGC  R: GAAGGTCCACGGGAAAGACAC  R: TTTTGCTTTTGTGCCAGAGGT  R: TTGTCACCAGCATCAGTCCC  R: GTCTACTGTCTCTAACGCTGCCTTG  R: TAAAACGCAGCTCAGTAACAGTCCG  R: TATTCCATGAGTTTCCGGTTGA  R: GATGCTATGTGTCACGTCTCTA  R: AAAGTGTCCATGTTTCAGCTTC  R: CGCCCTTGGAATGTATGTGGAGAG  R: GTTTAGGGCTGGGATGTTGATGAGG  R: GTTCCTCGTTCTCCACTCTCACATG  R: GCGAGGAACACTGAGATGTCTTGAG  R: TTACTAAGGAAGCGATGAAGCAGAAGG |
