# Supplementary material for: Therapeutic Mechanisms of Berberine to Improve the Intestinal Barrier Function via Modulating Gut Microbiota, TLR4/NF-κ B/MTORC Pathway and Autophagy in Cats
Source: Front Microbiol. 2022 Jul 22;13:961885. doi: 10.3389/fmicb.2022.961885 (PMC9354406; doi:10.3389/fmicb.2022.961885)
Supplement: Supplementary file 3 [file Data_Sheet_3.docx]

**Table S3. Antibodies for Western blot assay.**

| **Antibody** | **Vendor** | **Catalog No.** | **KD** |
| --- | --- | --- | --- |
| HRP anti-β-actin | Abclonal | AC026 | 43 |
| Anti-TLR4  Anti-NF-κB  Anti-mTOR  Anti-P-mTOR  Anti-GβL  Anti-Raptor  Anti-Rictor  Anti-LC3  Anti-Atg5  Anti-Atg7  Anti-p62  Anti-Slug  Anti-E-cadherin  Anti-N-cadherin | Beyotime  Beyotime  Cell Signaling Technology  Cell Signaling Technology  Cell Signaling Technology  Cell Signaling Technology  Cell Signaling Technology  Cell Signaling Technology  Cell Signaling Technology  Cell Signaling Technology  Wanlei  Cell Signaling Technology  Cell Signaling Technology  Cell Signaling Technology | AF8187  AF5327  2983  2974  3274  2280  2114  12741  12994  8558  WL02385  9585  3195  13116 | 120  65  289  289  37  150  200  14  55  78  62  30  135  140 |
