# Supplementary material for: Therapeutic Mechanisms of Berberine to Improve the Intestinal Barrier Function via Modulating Gut Microbiota, TLR4/NF-κ B/MTORC Pathway and Autophagy in Cats
Source: Front Microbiol. 2022 Jul 22;13:961885. doi: 10.3389/fmicb.2022.961885 (PMC9354406; doi:10.3389/fmicb.2022.961885)
Supplement: Supplementary file 4 [file Data_Sheet_4.pdf]

A Colonic mucosal damage score

1: Slight damage of surface epithelium;

2: Loss and injury of villus tip epithelial cells;

3: Mucosal vascular obstruction, hemorrhage and focal necrosis, villus loss of less than half;

4: Damage extends to more than half of the villi. Each group's colonic mucosal damage score.

B Typical illustrations

1

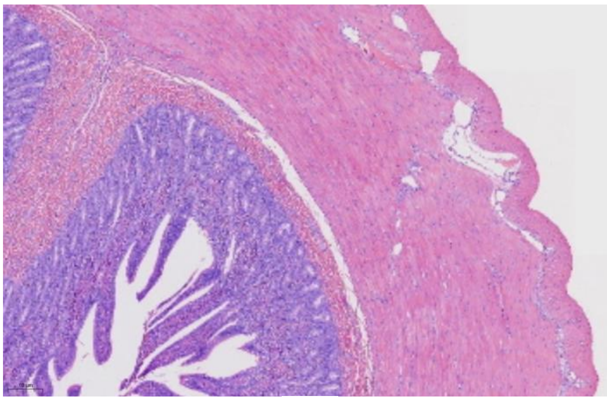

2

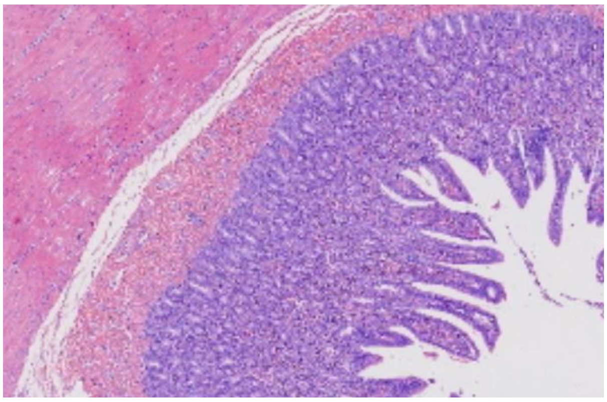

3

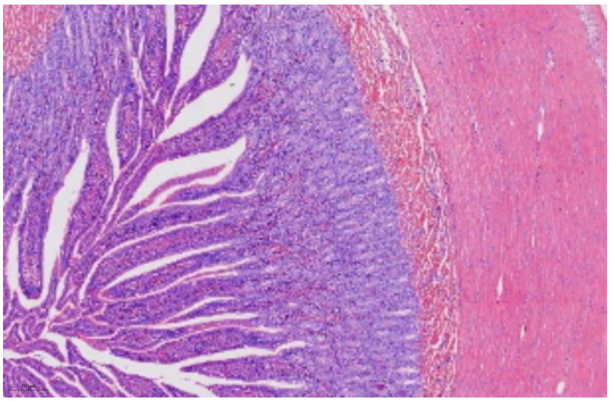

4

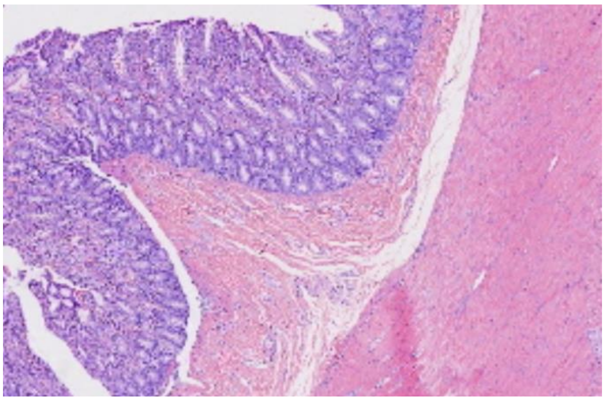

Supplementary Figure 1. **(A)** Histological assessment of the intestinal injury. The evaluation standard of the degree of tissue injury was a 0-4 scale. **(B)** Legends for each score.
