# Supplementary material for: Therapeutic Mechanisms of Berberine to Improve the Intestinal Barrier Function via Modulating Gut Microbiota, TLR4/NF-κ B/MTORC Pathway and Autophagy in Cats
Source: Front Microbiol. 2022 Jul 22;13:961885. doi: 10.3389/fmicb.2022.961885 (PMC9354406; doi:10.3389/fmicb.2022.961885)
Supplement: Supplementary file 5 [file Data_Sheet_5.pdf]

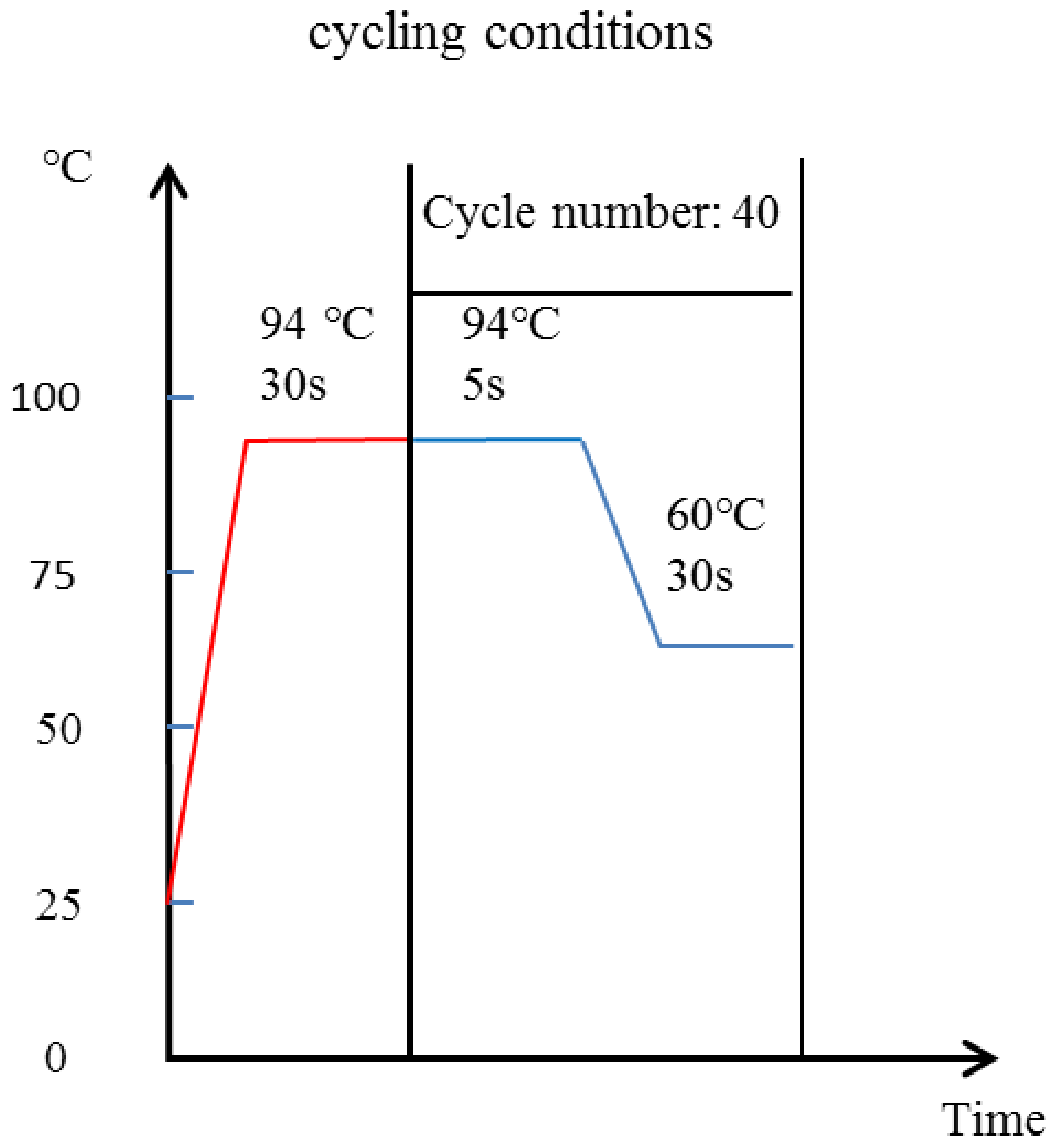

Supplementary Figure 2. The cycling conditions of RT-qPCR. Predenaturation at 94 ℃ for 30 s followed by 40 cycles of 94 ℃ for 5 s and annealing at 60 ℃ for 30 s.
