# Supplementary material for: Therapeutic Mechanisms of Berberine to Improve the Intestinal Barrier Function via Modulating Gut Microbiota, TLR4/NF-κ B/MTORC Pathway and Autophagy in Cats
Source: Front Microbiol. 2022 Jul 22;13:961885. doi: 10.3389/fmicb.2022.961885 (PMC9354406; doi:10.3389/fmicb.2022.961885)
Supplement: Supplementary file 6 [file Data_Sheet_6.pdf]

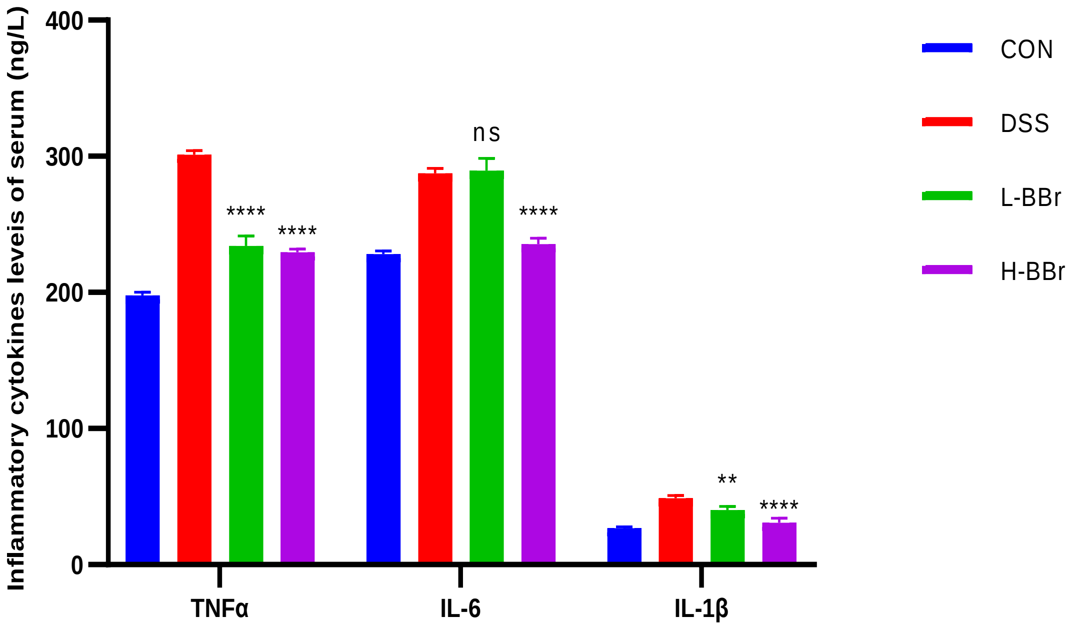

Supplementary Figure 3. BBr reduced the level of inflammatory cytokines. Serum level of IL-6, IL-1 β , and TNF- α . All data present as mean SD; n=3 per group. *P<0.05, **P<0.01, and ***P<0.001, ****＜0.0001, compared with the DSS group.
