# Supplementary material for: Therapeutic Mechanisms of Berberine to Improve the Intestinal Barrier Function via Modulating Gut Microbiota, TLR4/NF-κ B/MTORC Pathway and Autophagy in Cats
Source: Front Microbiol. 2022 Jul 22;13:961885. doi: 10.3389/fmicb.2022.961885 (PMC9354406; doi:10.3389/fmicb.2022.961885)
Supplement: Supplementary file 7 [file Data_Sheet_7.pdf]

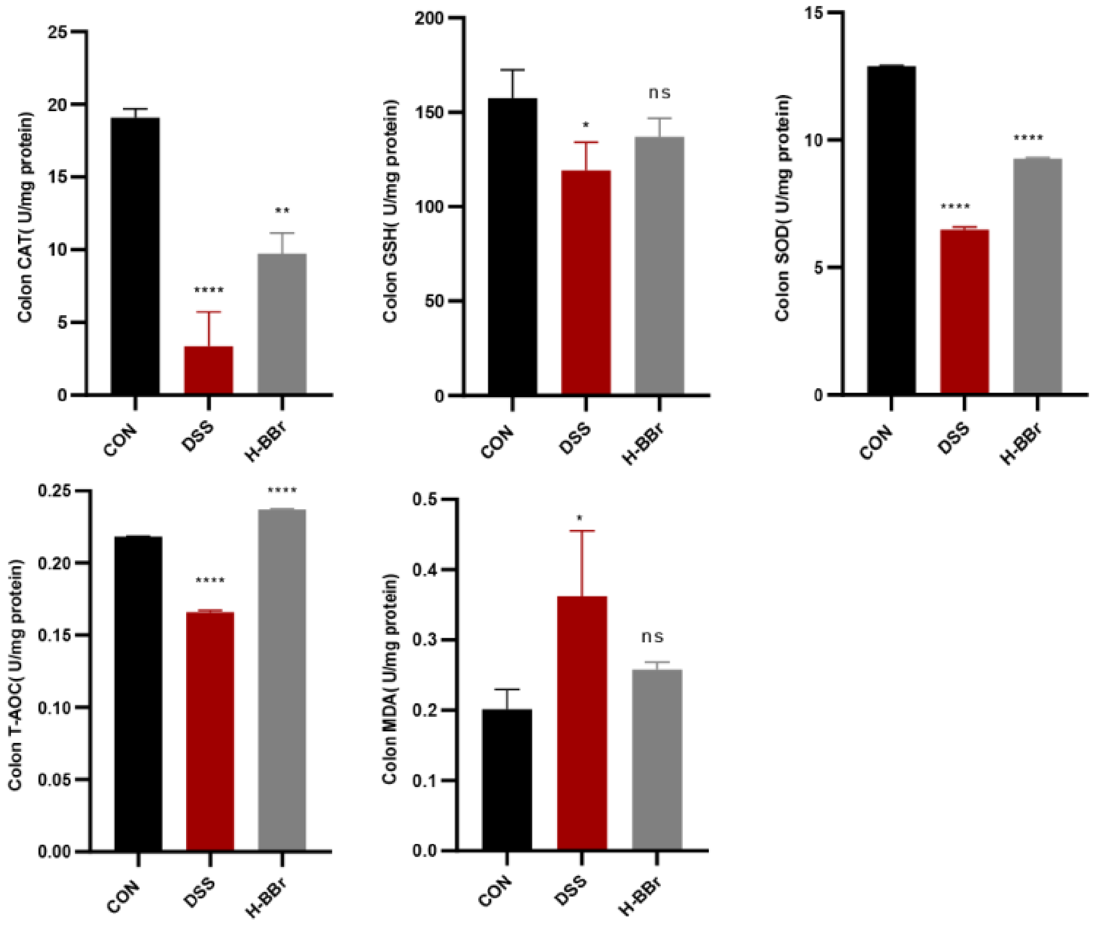

Supplementary Figure 4. BBr inhibited the oxidative stress responses in DSS-induced colitis. Colonic level of T-AOC, GSH, CAT, SOD, and MDA. All data present as mean SD; n=3 per group. *P<0.05, **P<0.01, and ***P<0.001,****＜0.0001, compared with the CON group.
