# Supplementary material for: Therapeutic Mechanisms of Berberine to Improve the Intestinal Barrier Function via Modulating Gut Microbiota, TLR4/NF-κ B/MTORC Pathway and Autophagy in Cats
Source: Front Microbiol. 2022 Jul 22;13:961885. doi: 10.3389/fmicb.2022.961885 (PMC9354406; doi:10.3389/fmicb.2022.961885)
Supplement: Supplementary file 8 [file Data_Sheet_8.pdf]

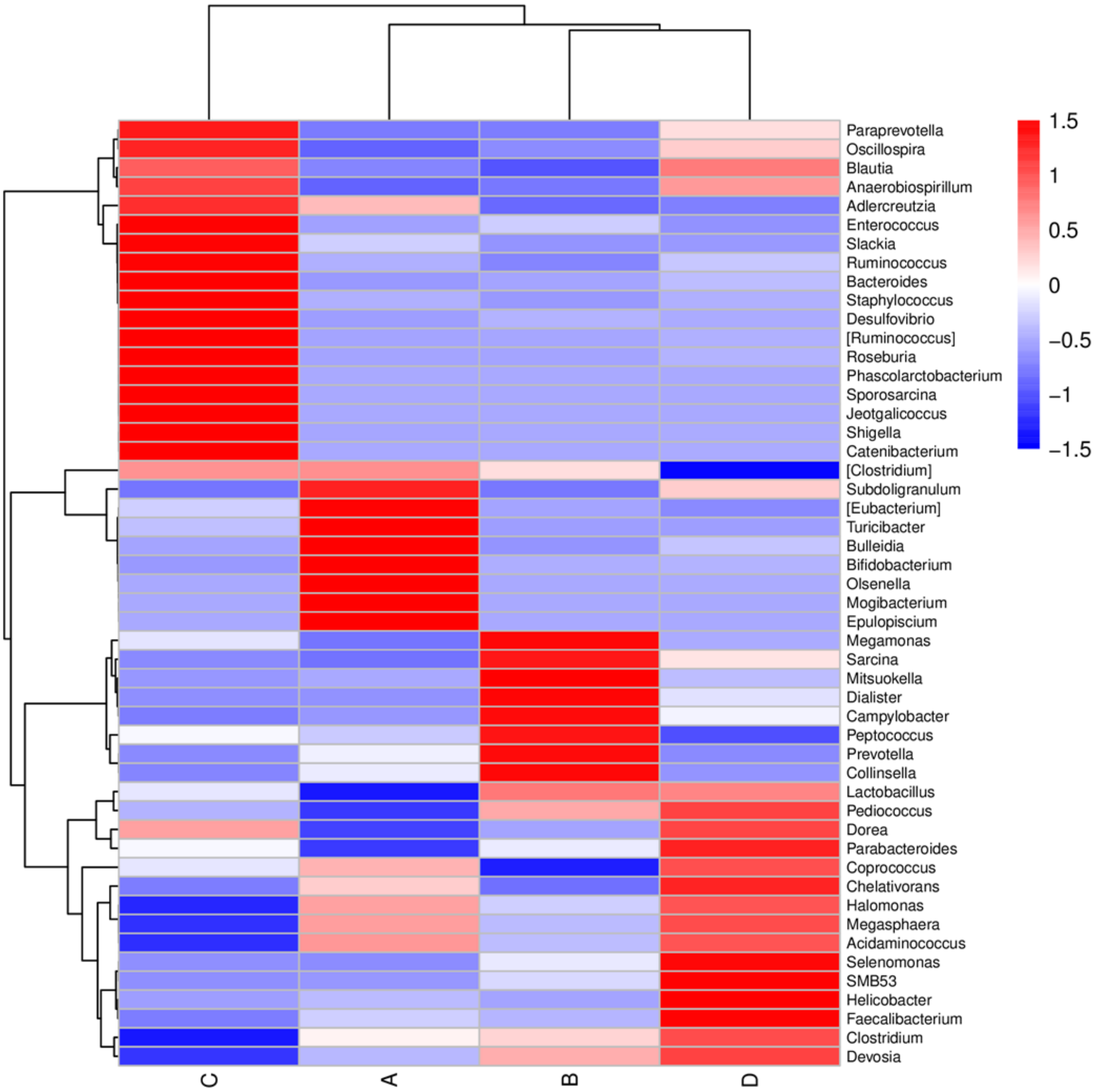

Supplementary Figure 5. Species composition heat map with genus level of species clustering. Different colors on the abscissa represented different groups and arranged species according to species clustering on the ordinate. Species with significant differences in abundance between groups were marked red, and the darker the color, the higher the abundance. A: CON group; B: DSS group; C: L-BBr group. D: H-BBr.
